# On the External Use of Opium in Inflammatory Diseases

**Published:** 1831-09

**Authors:** Robert Burn

**Affiliations:** Surgeon, Belford.


### OPIUM IN INFLAMMATION.

*On the External Use of Opium in Inflammatory Diseases.* By Robert Burn, Esq. Surgeon, Belford. Communicated by Dr. Bow.

TO DR. BOW, ALNWICK.

*Belford; 19th July,* 1831.

MY DEAR SIR,

I have very great satisfaction in being able to state that your plan of treating croup, and other inflammatory diseases, has succeeded with me most completely. In a disease so frightful and fatal as croup, I confess I was not bold enough at first to rely altogether on the means advised by you, but, in addition, applied leeches. However, since you informed me that you found leeching rather to retard than forward the cure, I have discarded them, and I agree with you that the liniment acts with more marked effect in the cases in which they have not been applied. All my cases, in which the liniment and calomel only were used, confirm the truth of the observations you make in the London Medical and Physical Journal for this month, viz. that the change from disease to health is so rapid, that there is no convalescent period. I sincerely congratulate you on this most decided improvement in our art, and enclose a few out of many cases which have come under my care.

I am, my dear sir, yours truly,

ROBERT BURN.

Case I. 25th May, 1831. Mary Pile, aged eleven months, has been ill for thirty-six hours, with difficulty of breathing, &c. The symptoms evidently becoming worse, I was sent for, and found the case one of decided croup. I ordered two drachms of liniment* to be rubbed on the breast and neck, and to be repeated at intervals of six hours; also two grains and a half of calomel every two hours, until the bowels were freely opened.

26th May. The child got better and better after each application of the liniment; the bowels have been freely opened.

27th. Perfectly well.

Case II. 16th June, 1831. — Sutherland, aged two years six months: breathing very difficult; skin hot; pulse rapid; cough frequent, with the peculiar croupy sound. Three drachms of liniment, and two grains of calomel every hour until it operate on the bowels.

17th. The child was immediately relieved by the liniment, but, as the parents were prejudiced in favor of leeches, four had been applied.

19th. Quite well.

Case III. 20th June, 1831. Mary Clarke, aged three years, has been ill for twenty-four hours of a supposed cold: the case, however, is decidedly one of croup, characterized by difficult and sonorous breathing, cough, &c. Three drachms of liniment.

Nine p.m. The child was immediately relieved by the liniment; indeed, as the mother said, before it was all applied. The breathing is again somewhat heavy, and the cough still croupy. Repeat the liniment, with a dose of calomel.

* The formula for this liniment is given by Dr. Bow, in our July number, p. 24.—Editor.

21st June. Has slept well all night; breathing relieved; cough easier. Repeat the liniment.

Evening. Continues better.

22d. Quite well.

Case IV. 2d July, 1831. — Crinkly, aged nine years six months: a most formidable case of croup, of three days' standing. The difficulty of breathing was most alarming; skin remarkably hot, and pulse exceedingly quick. Upwards of three drachms of liniment were immediately applied, and a full dose of calomel given.

3d. The relief from the liniment was almost instantaneous, but, as a child in the same family had died of the disease some time before, the parents could not be persuaded to trust to the liniment alone, so *they* applied six leeches, which bled freely. The calomel has acted on the bowels.

4th. The patient continues quite well, excepting some debility occasioned by the bleeding.

Case V. 11th July, 1831. — Robson, aged two years. This case was, with regard to symptoms, exactly similar to the last: it was seen at eleven P.M., and two drachms of the liniment were ordered to be occasionally applied to the neck and breast; calomel.

12th. The child was immediately relieved, and slept well during the night. The bowels have acted.

13th. Quite well.

